# Supplementary material for: Mechanism for inverted-repeat recombination induced by a replication fork barrier
Source: Nat Commun. 2022 Jan 10;13:32. doi: 10.1038/s41467-021-27443-w (PMC8748988; doi:10.1038/s41467-021-27443-w)
Supplement: Supplementary file 1 — Supplementary Information [file 41467_2021_27443_MOESM1_ESM.pdf]

Mechanism for inverted-repeat recombination induced by a replication fork barrier

a

PROBE	PRIMERS NAMES AND SEQUENCES	ANNEALING REGION	SIZE
A	Olea55 *GATAAGCTTCGTAACCGACAG*	*ade2*	710 bp
	Olea204 *GCTAGAAACTTGCGAAAGAGC*		
B	Olea203 *ATAATGGCGTTCGTTGTAATGG*	*ade2*	663 bp
	Olea200 *AGAAACAATCAGATTGATACAAGAC*		
C	Olea171b *ACTAAAGTAATCATGCTACGTACC*	*Ter* repeats	595 bp
	Olea177 *GTATGGTGCACTCCTCGAC*		
D	Olea175b *CACTCACACCATTCACACTC*	*his2*	540 bp
	Olea176 *GAAGTGACCCACGACCAAC*		
E	Olea201 *CGAGGATTTTTACTGAATTGTACG*	telomeric to *his2*	720 bp
	Olea202 *CATTATATGGGTATAAGAAGATGGC*		

b

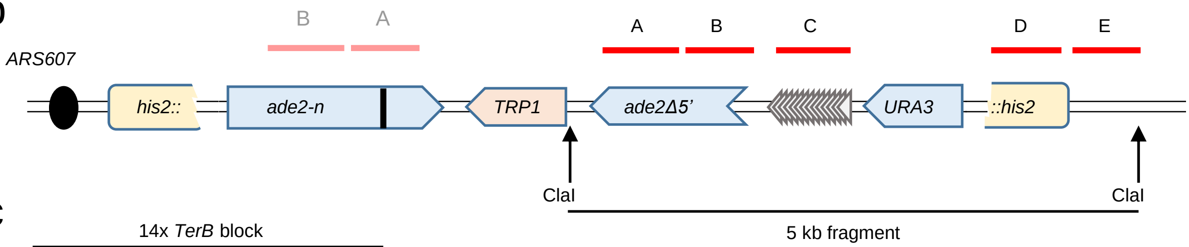

c

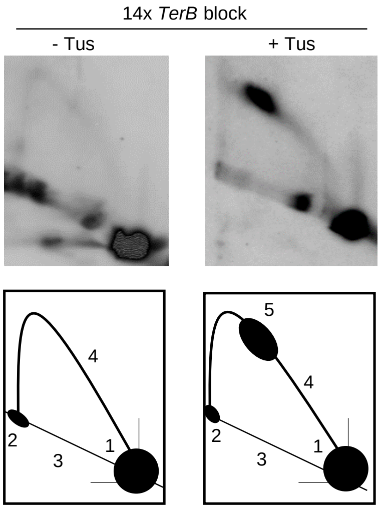

Supplementary Fig. 1. Two-dimensional gel analysis of replication intermediates. a Table of oligonucleotides used to generate radiolabelled probes for hybridization. **b** Schematic of the digestion fragment detected by 2D gel. Radiolabeled probes are represented by red lines and cover 3.2 kb of the digested 5 kb fragment. Probes A and B anneal to the two *ade2* repeats. After ClaI digestion, the *ade2-n* repeat is part of a 11.2 kb fragment which does not interfere with detection of the signal from the smaller 5 kb fragment containing the *Ter* repeats. **c** Interpretation of 2D gel analysis images. 1 = ClaI fragment before replication. 2 = ClaI fragment after almost complete replication. 3 = non specific linear DNA. 4 = arc of Y-shaped replication intermediates. 5 = fork stall corresponding to the position of the Tus/*Ter* barrier.

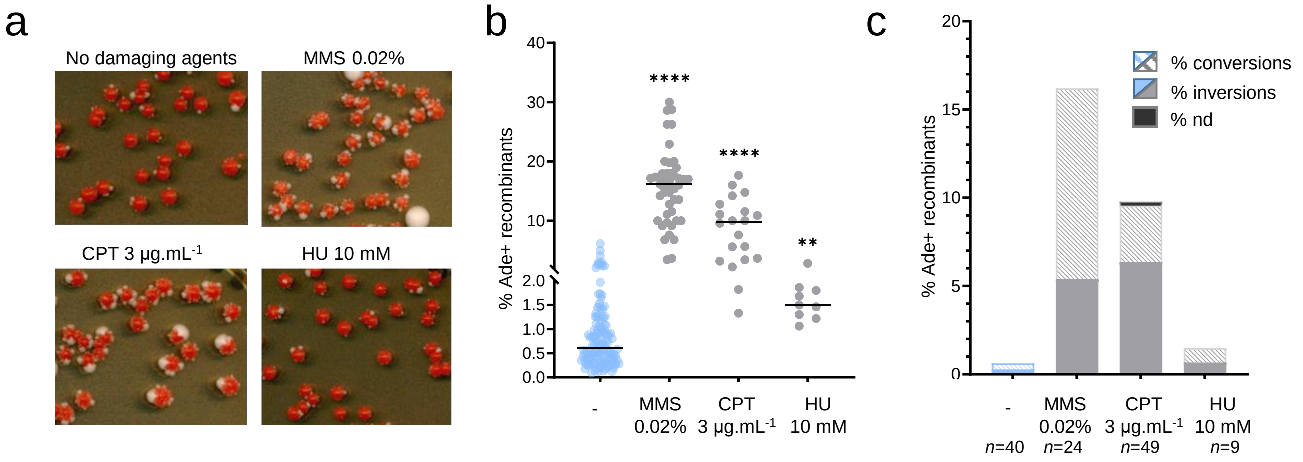

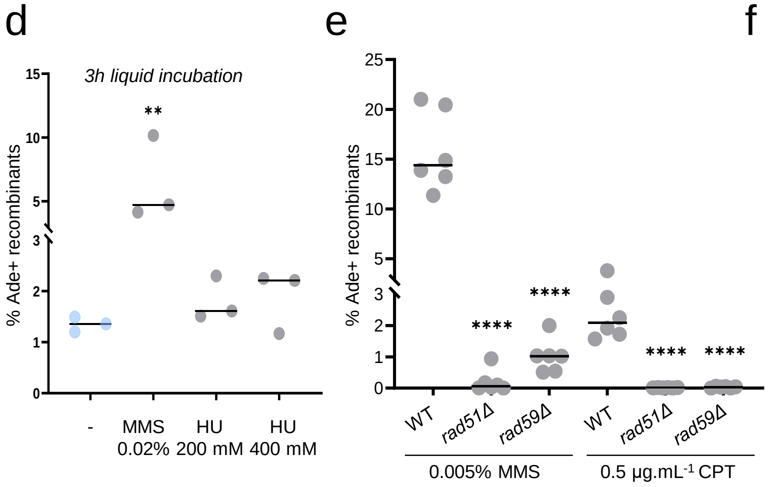

f

STRAIN	GENOTOXIC	MEDIAN [LL, UL]	# TESTED
WT	-	0.615 [0.51, 0.82]	126
	MMS 0.02 %	16.15 [13.55, 17.37]	46
	MMS 0.005 %	14.38 [11.36, 21.0]	6
	CPT 3 µg.mL	9.785 [3.66, 11.54]	20
	CPT 0.5 µg.mL	2.085 [1.57, 3.8]	6
	HU 10 mM	1.5 [1.22, 1.86]	9
rad51Δ	MMS 0.005 %	0.065 [0.0, 0.94]	6
	CPT 0.5 µg.mL	0.015 [0.0, 0.2]	6
rad59Δ	MMS 0.005 %	1.025 [0.51, 2]	6
	CPT 0.5 µg.mL	0.035 [0.0, 0.06]	6
WT	-	1.36 [1.2, 1.49]	3
	MMS 0.02 %	4.73 [4.15, 10.16]	3
	HU 200 mM	1.61 [1.51, 2.3]	3
	HU 400 mM	2.21 [1.17, 2.25]	3

Supplementary Fig. 2. Genome wide replication stress induces Rad51 and Rad59-dependent NAHR at inverted repeats. a Colonies form more white sectors (indicative of Ade^+ phenotype) on plates containing genotoxic agents inducing replication stress. **b** Ade^+ recombination frequencies without (blue data points) and with (grey data points) genotoxic agents. Concentrations from 2 mM to 150 mM HU were tested and showed comparable results. Black lines indicate medians. P-values were obtained on log transformed data by one-way Anova with a Boneferroni post-test and are relative to the NO genotoxic agent data with **** p-value <0.0001, ** p-value<0.005. **c** Distribution of NAHR events, with and without genotoxic agents, scored by PCR. *n* indicates the number of independent events examined for each strain. **d** Ade^+ recombination frequencies after 3 h liquid incubation with MMS and high concentrations of HU. The experiment was repeated 3 times with 9 independent cultures. One repeat is shown. P-values were obtained on log transformed data by one-way Anova with a Boneferroni post-test and are relative to the NO genotoxic agent data with ** p-value<0.005 **e** Ade^+ recombination frequencies with low concentrations of MMS and CPT in the WT and mutant strains. Black lines indicate medians. P-values, reported as stars when significant, are relative to the WT strain in the same condition and were obtained on log transformed data by one-way Anova with a Boneferroni post-test : **** p-value <0.0001. **f** Quantification of Ade^+ recombinants in the different strains in presence of genotoxic agents. >95% confidence intervals to the median are indicated as [LL, UL], where LL is the lower limit and UL is the upper limit. The orange line indicates quantifications from liquid incubations. **b-e** Exact p-values are reported in Supplementary Data 1. Source data are provided as a Source Data file.

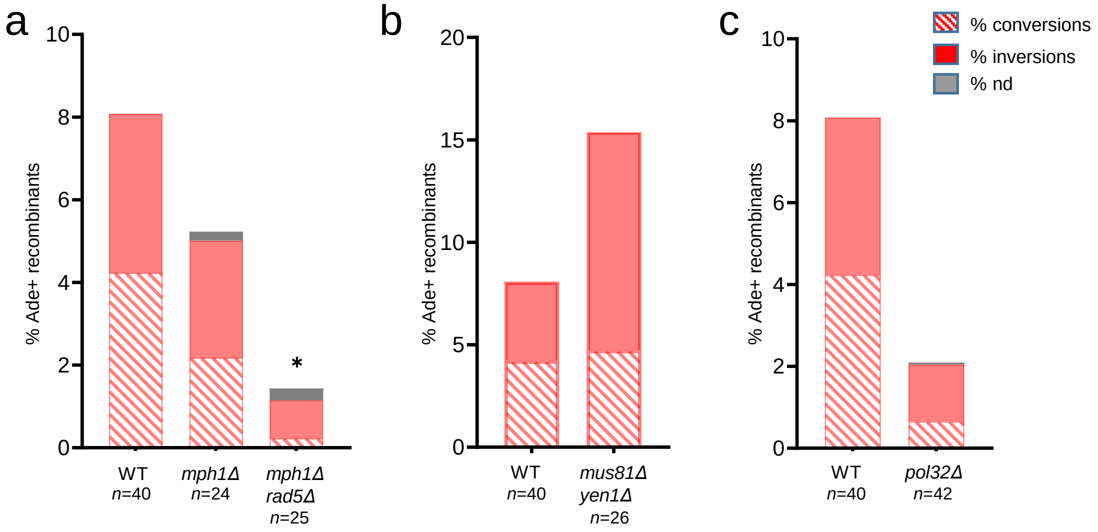

Supplementary Fig. 3. Distribution of NAHR events in WT and mutant strains. a Distribution of NAHR events in the WT, *mph1Δ* and *mph1Δ rad5Δ* strains, in response to the Tus/*Ter* block, scored by PCR**. b** Distribution of NAHR events in the WT and *mus81Δ yen1Δ strain*, scored by PCR. **c** Distribution of NAHR events in the WT and *pol32Δ* strains, scored by PCR**. a-c** nd= structure could not be determined by PCR. *n* indicates the number of independent Ade^{+} recombinants tested. P-values, reported as stars when significant, were obtained by Chi-Square test and are relative to the WT strain: * p-value <0.05. Exact p-values are reported in Supplementary Data 1 and source data are provided as a Source Data file.

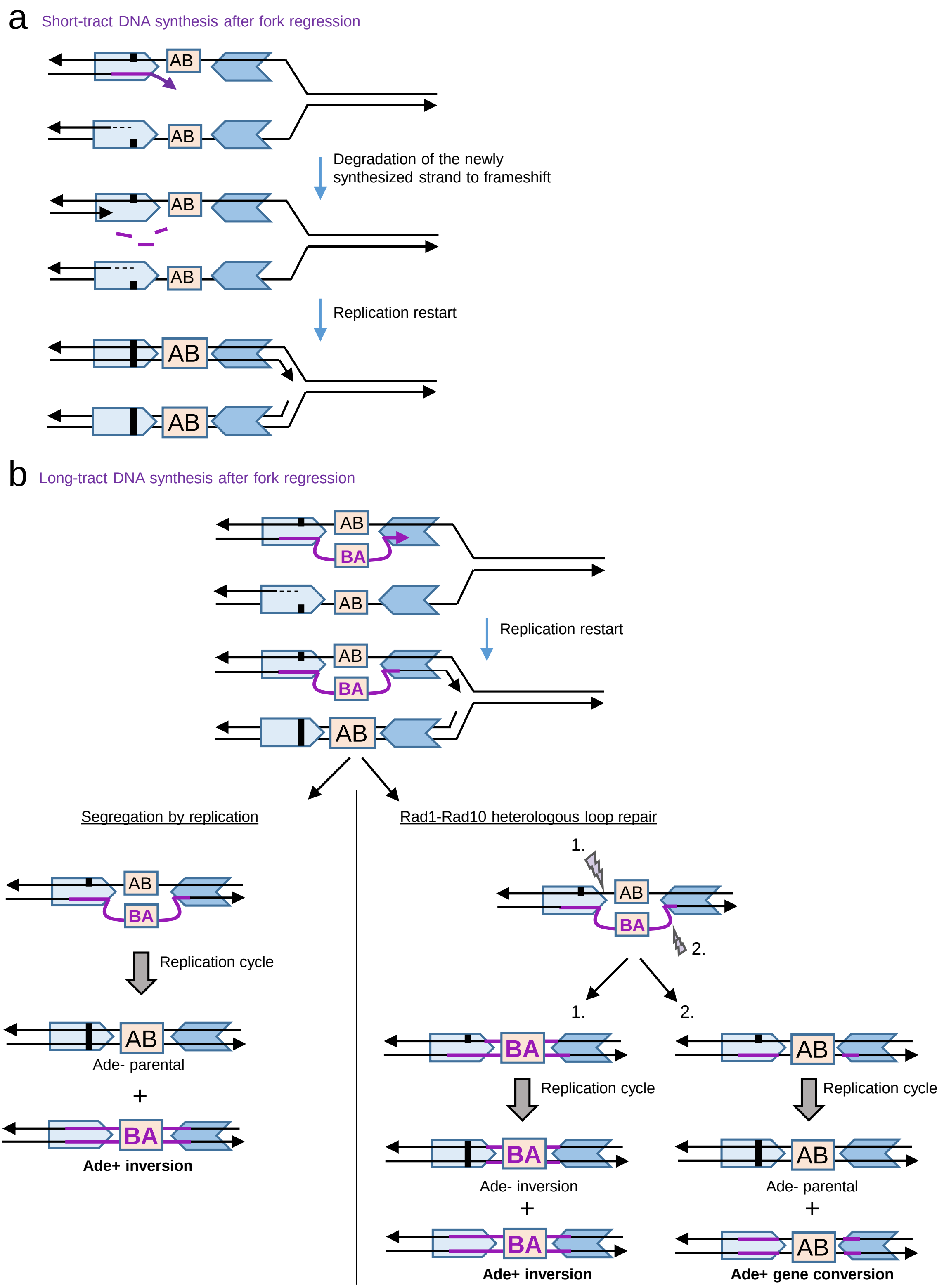

Supplementary Fig. 4. Alternative model for the late stages of replication-associated NAHR. a After fork regression, short-tract DNA synthesis forms a heterologous tail that is degraded by nucleases and no recombinant can form. **b** Long-tract DNA synthesis leads to the formation of a long heterologous loop between the repetitive sequences. The letters AB represent the orientation of the intervening sequence. Left panel shows resolution of the heterologous loop on the top arm by segregation of the unpaired strands at the next replication cycle. Right panel shows repair of the heterologous loop on the top arm by Rad1-Rad10 cleavage and DNA synthesis. Cleavage in positions 1 or 2 produces different outcomes. The bold black line indicates the +2 frameshift mutation in the long *ade2* cassette. Only cells without the frameshift are Ade^{+} and can be detected as recombinants. The mismatch repair pathway could also be involved in repairing the frameshift mutation.

Supplementary Table 1. Ade+ recombination frequencies

STRAIN	MEDIAN [LL, UL]	N^b	MEDIAN [LL, UL]	N^b
no *Ter*	0.47 [0.15, 0.78]	14	0.295 [0.06, 0.64]	10
perm *Ter*	0.87 [0.25, 2.4]	12	0.345 [0.16, 0.54]	12
WT = block *Ter*	0.615 [0.51, 0.82]	126	8.08 [6.96, 9.27]	127
rad51Δ	0.39 [0.24, 0.49]	54	0.32 [0.26, 0.68]	53
rad59Δ	0.46 [0.28, 0.72]	24	0.73 [0.45, 1.57]	18
rad51Δ rad59Δ	0.0 [0.0, 0.0]	6	0.0 [0.0, 0.0]	6
rad52Δ	0.0 [0.0, 0.0]	21	0.0 [0.0, 0.0]	22
rad51-II3A	0.205 [0.16, 0.28]	28	0.24 [0.14, 0.5]	22
rad52-R70A	0.145 [0.04, 0.21]	12	0.36 [0.18, 0.41]	12
rad57Δ	0.055 [0.01, 0.23]	24	0.1 [0.05, 0.24]	23
rad57Δ srs2Δ	0.235 [0.21, 0.42]	12	0.635 [0.4, 1.17]	12
srs2Δ	1.15 [0.96, 1.38]	28	47.61 [28.8, 60]	28
csm2Δ	1.33 [0.8, 1.89]	18	3.585 [2.41, 4.6]	18
rad54Δ	0.06 [0.02, 0.18]	12	0.31 [0.12, 1.15]	12
rad5Δ	0.9 [0.6, 1.3]	30	9.775 [5.9, 14.5]	30
mph1Δ	0.77 [0.52, 0.97]	12	5.225 [2.04, 7.0]	12
rad5Δ mph1Δ	0.24 [0.16, 0.65]	24	1.435 [1.06, 3.13]	24
mre11Δ	0.2 [0.04, 0.33]	12	2.295 [1.33, 3.67]	12
exo1Δ	0.85 [0.33, 1.25]	14	0.405 [0.25, 0.83]	14
sgs1Δ	1.065 [0.62, 1.28]	24	13.73 [8.51, 28.95]	20
rad1Δ	0.245 [0.13, 0.32]	12	1.0 [0.38, 1.65]	12
mus81Δ	0.695 [0.55, 0.88]	30	10.39 [7.2, 14.35]	30
mus81Δ yen1Δ	0.11 [0.09, 0.24]	18	15.45 [11.75, 17.20]	12
pol32Δ	0.265 [0.12, 0.54]	18	2.08 [1.21, 3.13]	24
rev3Δ	0.96 [0.48, 1.52]	12	12.88 [8.52, 21.36]	12
pol32Δ rev3Δ	0.395 [0.2, 1.04]	6	1.715 [0.9, 3.35]	12
pif1-m2	0.49 [0.34, 0.66]	12	7.29 [4.95, 10.76]	12
pif1-m2 dna2Δ	0.67 [0.41, 1.17]	12	2.14 [1.18, 3.9]	12

Blue data were obtained without Tus expression. Red data were obtained with Tus expression. All the mutant strains contain 14x*Ter* in the blocking orientation. For all strains, >95% confidence intervals to the median are indicated as [LL, UL], where LL is the lower limit and UL is the upper limit. N^b = number of independent colonies tested; perm= permissive orientation; block= blocking orientation.

Supplementary Table 2. Yeast strains

STRAIN	RELEVANT GENOTYPE	USE
LSY2002-9D	*his3::ade2Δ5'-TRP1-ade2-n*	Strain construction
LS4040	*his2::ade2-n-TRP1- ade2Δ5'*	Strain construction
LSY4577	*his2::ade2-n-TRP1- ade2Δ5' leu2::Gal-TUS-LEU2MX*	Recombination assay
LSY4579-1	*his2::ade2-n-TRP1- ade2Δ5'-**perm**14xTerB-URA3 leu2::Gal-TUS-LEU2MX*	Recombination assay
LSY4581-2	*his2::ade2-n-TRP1- ade2Δ5'-**block**14xTerB-URA3 leu2::Gal-TUS-LEU2MX*	Recombination assay
LSY4583	*his2::ade2-n-TRP1- ade2Δ5'-**block**14xTerB-URA3 leu2::Gal-TUS-LEU2MX **rad59**::LEU2*	Recombination assay
LSY4584	*his2::ade2-n-TRP1- ade2Δ5'-**block**14xTerB-URA3 leu2::Gal-TUS-LEU2MX **rad59**::LEU2 **rad51**::HIS3*	Recombination assay
LSY4585	*his2::ade2-n-TRP1- ade2Δ5'-**block**14xTerB-URA3 leu2::Gal-TUS-LEU2MX **rad51**::HIS3*	Recombination assay
LSY5127	*his2::ade2-n-TRP1- ade2Δ5'-**block**14xTerB-URA3 leu2::Gal-TUS-LEU2MX **rad52**::TRP1*	Recombination assay
LSY4618-1	*his2::ade2-n-TRP1- ade2Δ5'-**block**14xTerB-URA3 leu2::Gal-TUS-LEU2MX **rad51-II3A**-KanMX*	Recombination assay
LSY5122	*his2::ade2-n-TRP1- ade2Δ5'-**block**14xTerB-URA3 leu2::Gal-TUS-LEU2MX **rad57**::LEU2*	Recombination assay
LSY5134	*his2::ade2-n-TRP1- ade2Δ5'-**block**14xTerB-URA3 leu2::Gal-TUS-LEU2MX **srs2**::TRP1*	Recombination assay
LSY5013-12C	*his2::ade2-n-TRP1- ade2Δ5'-**block**14xTerB-URA3 leu2::Gal-TUS-LEU2MX **srs2**::TRP1 **rad57**::LEU2*	Recombination assay
LSY5123	*his2::ade2-n-TRP1- ade2Δ5'-**block**14xTerB-URA3 leu2::Gal-TUS-LEU2MX **csm2**::KanMX*	Recombination assay
LSY5128-1	*his2::ade2-n-TRP1- ade2Δ5'-**block**14xTerB-URA3 leu2::Gal-TUS-LEU2MX **rad54**::LEU2*	Recombination assay
LSY4459-1	*his2::ade2-n-TRP1- ade2Δ5'-**block**14xTerB-URA3 leu2::Gal-TUS-LEU2MX **rad5**::URA3*	Recombination assay
LSY5130	*his2::ade2-n-TRP1- ade2Δ5'-**block**14xTerB-URA3 leu2::Gal-TUS-LEU2MX **mph1**::KanMX*	Recombination assay
LSY5132	*his2::ade2-n-TRP1- ade2Δ5'-**block**14xTerB-URA3 leu2::Gal-TUS-LEU2 **mph1**::KanMX **rad5**::URA3*	Recombination assay
LSY5124-1	*his2::ade2-n-TRP1- ade2Δ5'-**block**14xTerB-URA3 leu2::Gal-TUS-LEU2MX **mre11**::LEU2*	Recombination assay
LSY4620	*his2::ade2-n-TRP1- ade2Δ5'-**block**14xTerB-URA3 leu2::Gal-TUS-LEU2MX **sgs1**::HIS3*	Recombination assay
LSY4621-1	*his2::ade2-n-TRP1- ade2Δ5'-**block**14xTerB-URA3 leu2::Gal-TUS-LEU2MX **exo1**::KanMX*	Recombination assay
LSY5135	*his2::ade2-n-TRP1- ade2Δ5'-**block**14xTerB-URA3 leu2::Gal-TUS-LEU2MX **rad1**::LEU2*	Recombination assay
LSY5129	*his2::ade2-n-TRP1- ade2Δ5'-**block**14xTerB-URA3 leu2::Gal-TUS-LEU2MX **mus81**::KanMX*	Recombination assay
LSY5133-1	*his2::ade2-n-TRP1- ade2Δ5'-**block**14xTerB-URA3 leu2::Gal-TUS-LEU2MX **mus81**::KanMX **yen1**::HIS3*	Recombination assay
LSY4592-1	*his2::ade2-n-TRP1- ade2Δ5'-**block**14xTerB-URA3 leu2::Gal-TUS-LEU2MX **pol32**::KanMX*	Recombination assay
LSY5125-1	*his2::ade2-n-TRP1- ade2Δ5'-**block**14xTerB-URA3 leu2::Gal-TUS-LEU2MX **pol32**::KanMX **rev3**::HIS3*	Recombination assay
LSY5126	*his2::ade2-n-TRP1- ade2Δ5'-**block**14xTerB-URA3 leu2::Gal-TUS-LEU2MX **rev3**::HIS3*	Recombination assay
LSY5067-15	*his2::ade2-n-TRP1- ade2Δ5'-**block**14xTerB-URA3 **bar1**::HYG*	2D gel analysis
LSY5060-4	*his2::ade2-n-TRP1- ade2Δ5'-**block**14xTerB-URA3 leu2::Gal-TUS-LEU2MX **bar1**::HYG*	2D gel analysis
LSY5075	*his2::ade2-n-TRP1- ade2Δ5'-**block**14xTerB-URA3 leu2::Gal-TUS-LEU2MX **rad51**::HIS3 **bar1**::HYG*	2D gel analysis
LSY5062	*his2::ade2-n-TRP1- ade2Δ5'-**block**14xTerB-URA3 leu2::Gal-TUS-LEU2MX **rad59**::LEU2 **bar1**::HYG*	2D gel analysis

All strains are haploids derived from W303 (*leu2-3,112 trp1-1 can1-100 ura3-1 his3-11,15)*, *ade2::hisG* and *RAD5* unless otherwise indicated.

Supplementary References.

1 Gnügge, R., Liphardt, T. & Rudolf, F. A shuttle vector series for precise genetic engineering of Saccharomyces cerevisiae. *Yeast* **33**, 83-98, doi:10.1002/yea.3144 (2016).

2 Liberi, G., C. Cotta-Ramusino, M. Lopes, J. Sogo, C. Conti, A. Bensimon, and M. Foiani. 2006. "Methods to study replication fork collapse in budding yeast." *Methods Enzymol* 409:442-62. doi: 10.1016/S0076-6879(05)09026-9.
