# Supplementary material for: Mechanism for inverted-repeat recombination induced by a replication fork barrier
Source: Nat Commun. 2022 Jan 10;13:32. doi: 10.1038/s41467-021-27443-w (PMC8748988; doi:10.1038/s41467-021-27443-w)
Supplement: Supplementary file 3 — Description of Additional Supplementary Files [file 41467_2021_27443_MOESM3_ESM.pdf]

Description of Additional Supplementary Files

Supplementary Data 1

Description: Specific p-values for data shown in Figures 1-4 and Supplementary Figs. 2.
